# Cholesteatoma of external auditory canal: a case report

**DOI:** 10.1016/S1808-8694(15)31291-X

**Published:** 2015-10-20

**Authors:** Fábio D. Zanini, Everton S. Ameno, Sidney O. Magaldi, Ruben A. Lamar

**Affiliations:** 1 Otorhinolaryngologist, Hospital Regional de São José Homero de Miranda Gomes – São José/SC; Hospital Infantil Joana de Gusmão – Florianópolis/SC; Fundaçã o Catarinense de Educaçã o Especial São José/SC; 2 Otorhinolaryngologist, Head of the Service of Otorhinolaryngology, Hospital da Lagoa – Rio de Janeiro/RJ; 3 Physician, Service of Otorhinolaryngology, Hospital da Lagoa – Rio de Janeiro/RJ; 4 Physician, Service of Otorhinolaryngology, Hospital da Lagoa – Rio de Janeiro/RJ

**Keywords:** cholesteatoma, external auditory canal, otorrhea

## Abstract

The authors present a case of cholesteatoma of external auditory canal (CEAC) with extensive invasion of mastoid; ossicle chain and tympanic membrane remained intact. The only symptom was chronic otorrhea. Diagnosis was based on clinical elements and CT scan was used to measure pathology and program surgery. Treatment was modified radical mastoidectomy associated with meatoplasty. Due to the insidious character of CEAC and the proximity with important structures of the external auditory canal, it must be always considered in differential diagnosis for lesions of external auditory canal. This case report intended to review clinical and surgical aspects of treatment of CEAC and present our approach in a case with severe lesions.

## INTRODUCTION

Cholesteatoma of the external auditory canal (CEAC) is a rare condition affecting especially elderly people. The progress of the disease is slow and the symptoms are not too evident. This leads to late diagnosis, with progressive bone lysis affecting important circumjacent structures^1^, ^2^.

The authors present a case of CEAC with extensive invasion of the mastoid, exposing the lateral sinus and the dura. Hearing acuity and structures of the tympanic cavity were unaffected.

We discuss the possible causes and treatment of choice.

## LITERATURE REVIEW

In clinical practice of ear diseases, cholesteatoma occurs on the tympanum-mastoid segment. It rarely originates from the external auditory canal (EAC), whose incidence is about 0.1-0.5% in new patients with ear problems^1^, ^2^, ^3^.

In 1850^2^, ^4^, Toynbee was the first author describing that cholesteatoma originates from the external auditory canal as “epidermal sheets”. Up to 1980, CEAC and keratosis obturans (KO) were considered different presentations of the same disease. Pipergerdes et al.^2^ described CEAC and keratosis obturans (KO) as two distinct clinical-pathological processes: KO as keratin accumulation in the EAC; and CEAC as bone erosion resulting from squamous tissue on a specific spot of the EAC^2^.

In CEAC, lateral epithelium desquamation is affected. Keratin debris are blocked, causing local erosion and bone lysis, which can be severe^2^, ^5^. Holt^2^, ^3^, ^6^, ^7^ listed situations in which the disease can be diagnosed: postoperative period of ear surgery; EAC trauma; EAC obstruction – due to osteoma or stenosis, for instance; idiopathic – possibly, in these cases, local periostitis may originate the process of hyperkeratosis.

Diagnosis is made through physical examination. There is otalgia, otorrhea, hearing acuity is unaffected; otoscopy shows an intact tympanic membrane and erosion on a distinct spot of the external auditory canal^4^, ^5^, ^8^. Otorhinolaryngologists must be aware that cholesteatoma may affect circumjacent structures (lateral sinus, facial nerve, posterior cranial fossa). Therefore, a CT scan is recommended for all patients^1^, ^2^, ^4^.

Differential diagnosis for CEAC should include necrotizing external otitis, tumors and KO. The latter is more frequent in young adults presenting severe otalgia and bilateral sensorial hearing loss. Differently from CEAC, which usually affects older patients, KO is unilateral, there is otorrhea, chronic pain and hearing acuity is preserved. In KO, the EAC has a keratin plug; otoscopy, following keratin plug removal, shows stenosis, redness and granulation tissue. In CEAC, there is an epidermal diverticulum on the lowest part of the canal, with epidermal debris and otorrhea; the other parts of the EAC are normal^1^, ^2^, ^3^.

The treatment of CEAC may be clinical or surgical. The former is carried out through local flushing and topic antibiotics. The latter is through resection of the cholesteatoma and necrotic bone^5^, ^6^, ^7^, ^8^.

Clinical treatment is indicated whenever the lesion is limited and there is no otalgia, in cases that surgery is contraindicated, in patients refusing surgical procedures and, when observation without treatment is an option to evaluate progression^2^, ^6^.

Surgical treatment is indicated in the following situations: chronic pain (despite treatment), frequent infection (there is risk for bacterial resistance), facial palsy or chronic dizziness, progression during follow-up, involvement of hypotympanum, jugular or mastoid (showed in CT scans), diabetes mellitus or immunosuppression (predisposition to necrotizing external otitis)^2^, ^4^, ^6^, ^9^.

## CASE REPORT

Patient A. F., male, 59 years old. Visited the otorhinolaryngological outpatient facility showing otorrhea, otalgia and ear fullness on the right for ten (10) years. Otoscopy on his right ear showed epidermal debris in the canal. Following debris removal, the tympanic membrane was intact, and there was severe erosion of the posteroinferior quadrant of the EAC. Otoscopy of the left ear was normal. Bilateral pure tone audiometry was normal.

**Figure 1:**
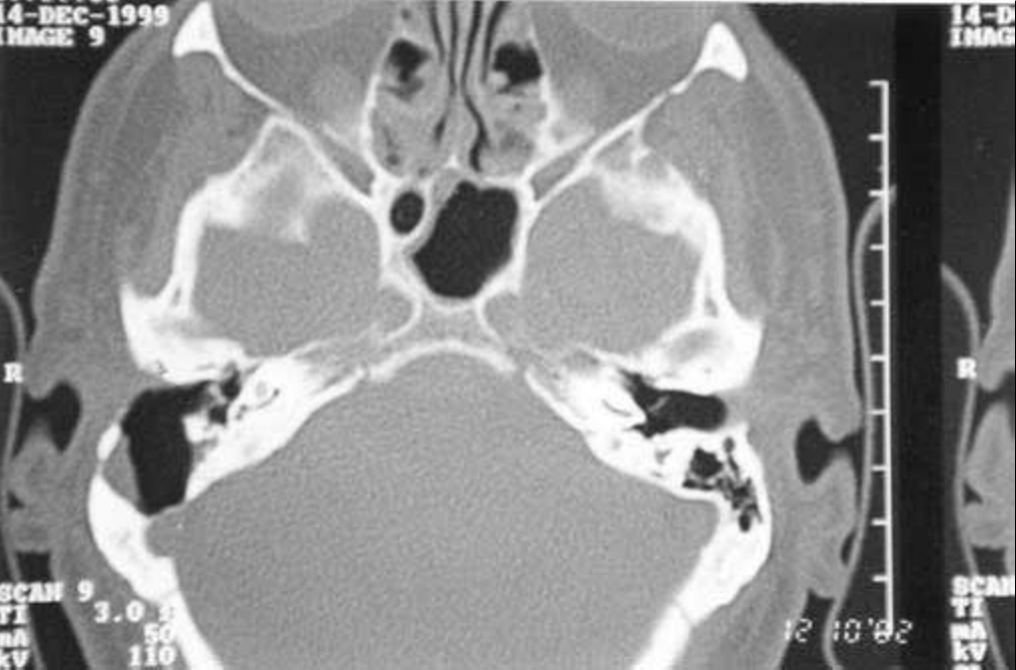
Temporal bone axial CT scan showing contact of the cholesteatoma's capsule with sigmoid sinus and meninges.

We suspected of CEAC and requested a CT scan of temporal bones. CT scan showed erosion of the posterior wall of the EAC, mastoid involvement and exposure of the lateral sinus and meninges (Figure 1).

**Figure 2:**
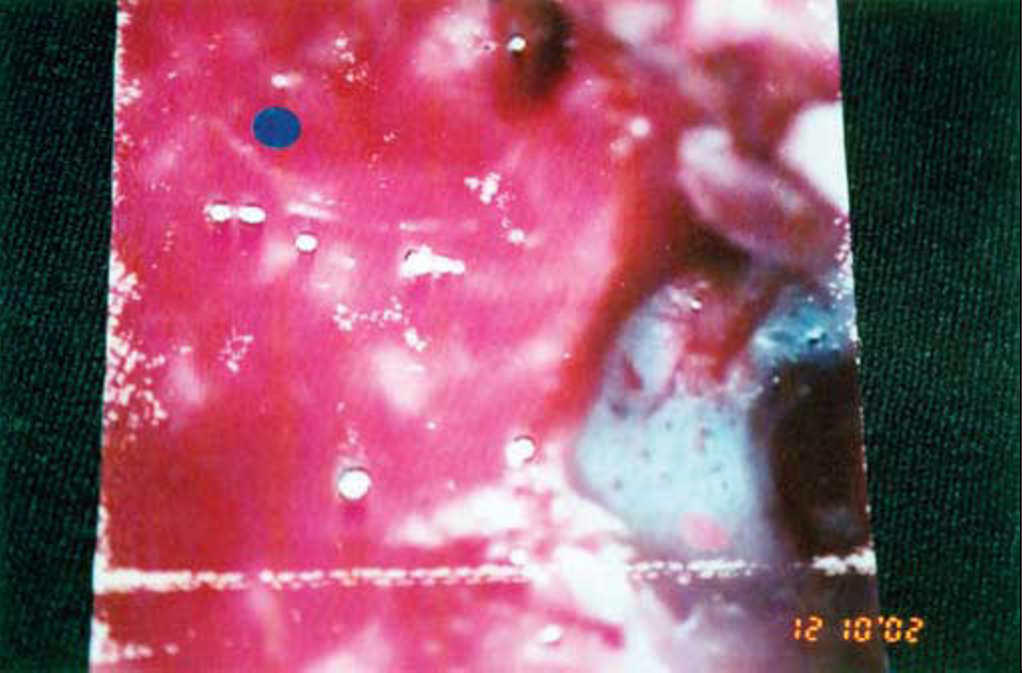
Transoperative picture of modified radical mastoidectomy showing intact TM and CEAC affecting the mastoid.

Transoperative observation revealed erosion of the entire posterior wall of the EAC, except for a thin bone band close to the tympanic ring (Figure 2). The tympanic membrane, ossicle chain and facial nerve canal were preserved.

We conducted modified radical mastoidectomy type I plus meatoplasty. The patient showed good outcomes and normal hearing, without recurrences, in a postoperative period of two years.

## DISCUSSION

CEAC, due to its slow and insidious progression, may severely affect structures of the middle ear at the time of presentation. In our case report, the lesion was severe at first presentation, and we were not able to visualize its limits through otoscopy. CT studies were important to determine the severity of the disease and involvement of other structures; therefore, CT scan is indicated whenever CEAC is suspected.

The disease had possibly appeared spontaneously, since the patient did not have any predisposing factor to develop CEAC.

Mastoid invasion is one of the indications for surgery. After the patient agreed to undergo surgery, we performed modified radical mastoidectomy and wide meatoplasty; thus, we could achieve good control of the cavity during the postoperative period.

We were able to preserve the tympanic cavity and its structures; therefore, the surgical procedure was able to cure and preserve the patient's hearing acuity. It is worth mentioning that, in spite of the increased progression of the disease, there was no involvement of the facial nerve, inner ear or nerves emerging from the jugular foramen.

## CLOSING REMARKS

Otorhinolaryngologists must suspect of CEAC in cases of chronic otorrhea and intact tympanic membrane. After diagnosis of CEAC, CT scans of the temporal bone must be performed in all patients to evaluate the real severity of the disease and involvement of circumjacent structures (jugular bulb, cranial nerves and structures of the tympanic cavity). In most cases, the treatment of choice is surgery, whose main purpose is to eradicate the lesion and, if possible, preserve the patient's hearing acuity. The modified radical mastoidectomy is the most common surgical procedure carried out in these cases.
